# “A Tale Preserved in a Museum”: The Long-Awaited Discovery of *Genitocotyle necromnemos* n. sp. (Trematoda: Opecoelidae) from the Cardinal Fish *Apogon imberbis* (Linnaeus) in the Western Mediterranean

**DOI:** 10.1007/s11686-025-01067-2

**Published:** 2025-07-14

**Authors:** Fatima-Zohra Zedam, Chahinez Bouguerche, Fadila Tazerouti

**Affiliations:** 1 Faculté des Sciences Biologiques, Département d’Écologie et Environnement, Laboratoire de Biodiversité et Environnement, Interactions - Génomes, Université des Sciences et de la Technologie Houari Boumediene (U.S.T.H.B), BP 32, El Alia Bab Ezzouar, Alger, Algeria; 2 Department of Zoology, Swedish Museum of Natural History, Box 50007, 104 05 Stockholm, Sweden

**Keywords:** Algeria, Apogonidae, Atlantic Ocean, Digenea, Madeira, Parasite

## Abstract

**Purpose:**

Opecoelids, a diverse group of digenean trematodes, pose a taxonomic challenge due to morphological similarities. The genus *Genitocotyle* is little studied with only five recognized species. Recent phylogenetic advancements have refined Opecoelidae classification, recognizing 15 subfamilies, including opecoelins, distinguished by the absence of a cirrus sac and a canalicular seminal receptacle. Within this subfamily, the genus *Genitocotyle* remains understudied, with only five recognized species. The aim of this study is to describe a new species and to clarify a long-standing taxonomic ambiguity.

**Methods:**

Two historical specimens of *Genitocotyl*e from *Apogon imberbis* off Madeira, preserved in the Natural History Museum, London, were examined alongside newly collected specimens from Algerian waters. Comparative morphological analyses, including illustrations and morphometric measurements, were conducted.

**Results:**

*Genitocotyle necromnemos* n. sp. is described, differing from its congeners by its larger body size, the more numerous eggs, the extended post-testicular region, the seminal vesicle restricted to the forebody, the extension of the vitellarium and the shape of the ovary. This new species designation clarifies the identity of some museum specimens, undescribed since their collection in 1951.

**Conclusion:**

This study highlights the importance of museum collections for the clarification of taxonomic uncertainties and the expansion of knowledge about the biodiversity of trematodes. The result contribute the systematics of opecoelines and emphasise the ecological and biogeographical importance of *Genitocotyle* spp. in marine fish hosts.

## Introduction

The opecoelids belong to one of the most taxonomically confusing groups of digeneans [1]. The family Opecoelidae Ozaki, 1925 comprises a diverse group of trematodes that parasitize marine and freshwater fishes [2]. Although the opecoelids are a large and evolutionarily derived group, the morphology of the adults is not very diverse or specialized [3]. Since the early 1980 s, the organization of Opecoelidae has been primarily based on classification hypothesis with four-subfamilies (see Martin [4]), with a lack of an appropriate subfamily classification [5]. In recent years, a new subfamily classification for Opecoelidae has been developed that reflects the phylogeny and provides practical taxonomic subdivisions. The proposed classification includes at least 15 subfamilies, based on phylogenetic relationships, adult morphology, and life-cycle ecology [3, 6–8].

The subfamily Opecoelinae Ozaki, 1925 is defined for species with reduced or absent cirrus sac and lacking a canalicular seminal receptacle [1]. It currently comprises 22 recognized genera [9]. Despite the general morphological uniformity, genera within this subfamily differ primarily by variations in the gut structure of the respective species (many exhibit configurations other than blind caeca), the shape and characteristics of the ventral sucker (e.g. the presence of papillae or a stalked form), and the distribution of the vitelline follicles [1]. Three genera, *Anisoporu*s Ozaki, 1928, *Opecoeloides* Odhner, 1928 and *Genitocotyle* Park, 1937, in which the corresponding species are characterised by an unusual accessory sucker located between the genital atrium and the ventral sucker. The main differences between these genera lie in the termination of the intestinal caeca. In *Anisoporus* spp. the caeca open through a single anus, whereas in *Opecoeloides* spp. they connect to the base of the excretory vesicle and form a uroproct. In contrast, the caeca of *Genitocotyle* spp. are blind-ended [10].

To date, *Genitocotyle* comprises only five species [11]: (i). *G. acirru*s Park, 1937, the type species described from the stomach and upper intestine of the redtail surfperch *Amphistichus rhodoterus* (Agassiz) (Embiotocidae) off California (Eastern Central Pacific) [12]; (ii) *G. atlantica* Manter, 1947, described from the intestine of various fish families, off Florida (Western-Central Atlantic) [13]; (iii) *G. cablei* Park, 1937, found in the intestine of the ocellated flounder *Ancylopsetta quadrocellata* Gill (Paralichthyidae), off Dog Island Reef, Florida (Western-Central Atlantic) [14]; (iv) *G. heterostichi* Montgomery, 1957 described from the intestine of the giant kelpfish *Heterostichus rostratus* Girard (Clinidae) off La Jolla, California (Eastern Central Pacific) [15]; and (v) *G. mediterranea* Bartoli, Gibson and Riutort, 1994 described from the intestine of the ocellated wrasse *Symphodus ocellatus* (L.) (Labridae) off Corsica, France (Western Mediterranean) [10].

*Genitocotyle* spp. are distinguished by several morphological criteria, including the distribution of vitelline follicles, the extent of their yolk fields and the dimensions and shape of specific organs such as ovary and testes. Additional distinguishing features include the number of eggs, the presence of elongated seminal vesicles, and the length of the post-testicular region. These characteristics are sufficient to distinguish the species within this genus and help to characterise new species within this genus [10, 12–19]

Only one species of *Genitocotyle* has been recorded from the Mediterranean Sea—*G. mediterranea* [10]. However, two *Genitocotyle* specimens from a cardinal fish, *Apogon imberbis* (Apogonidae) (originated from Madeira, but died in 1951 in the aquarium of London Zoo), preserved in the Natural History Museum, London (NHMUK), showed similarities with *G. mediterranea*, but had notable differences, such as a larger size, more numerous eggs, a longer post-testicular zone and a seminal vesicle apparently restricted to the forebody [10]. Their conspecificity with *G. mediterranea* remained uncertain and required fresh material for confirmation [10]. Herein, we address the long-standing enigma of these digeneans. Although they were suspected and published as potentially new species, they remained undescribed due to limitations. We formally describe them as a new species of *Genitocotyle* based on the study of the museum specimens and freshly collected specimens from *A. imberbis* off Algeria.

## Materials and Methods

From 2020 to 2024, a total of 218 specimens of *A. imberbis* were obtained from local fishermen in Dellys and Tamentfoust, Algeria, Western Mediterranean. Fish were transferred to the laboratory immediately after capture and identified using keys [20]. The gastrointestinal tract was removed and examined for trematodes using a stereomicroscope (Carl Zeiss^™^ 2000 Stereomicroscope, Germany) for the presence of helminths. Trematodes were removed alive using fine dissecting needles and heat-killed. Due to the need for detailed morphological comparison, all *Genitocotyle* specimens were flattened during preparation and then fixed in 70% ethanol with Bouin’s fluid [10]. While this method is not ideal for preservation [21], it was necessary to facilitate accurate comparison of key morphological features across species. Whole mounts were stained with boracic carmine, dehydrated through a graded ethanol series (70, 96 and 100%), cleared in clove oil, and mounted in Canada balsam [22]. Drawings were made with the aid of a Leitz microscope (USTHB, Algeria) and a Nikon Eclipse i80 microscope with DIC (differential interference contrast) (SMNH, Sweden) equipped with a drawing tube, scanned and redrawn with Adobe Illustrator (CS5). Measurements of whole-mounts are in micrometres and indicated as the range followed by mean values in parentheses. Additionally, two specimens of *Genitocotyle* sp. from the intestine of *A. imberbis* off Madeira (NHMUK Coll. No. 1980.7.17.228) [10] were examined for comparison. To compare morphological and anatomical features across *Genitocotyle* spp., figures in the global literature were extracted from published PDF files following Bouguerche *et al*. [23]. The following abbreviations are used: SMNH, Swedish Museum of Natural History, Stockholm, Sweden; NHMUK, Natural History Museum, London, UK.

## Results

Family Opecoelidae Ozaki, 1925

Subfamily Opecoelinae Ozaki, 1925

Genus *Genitocotyle* Park, 1937

**Fig. 1:**
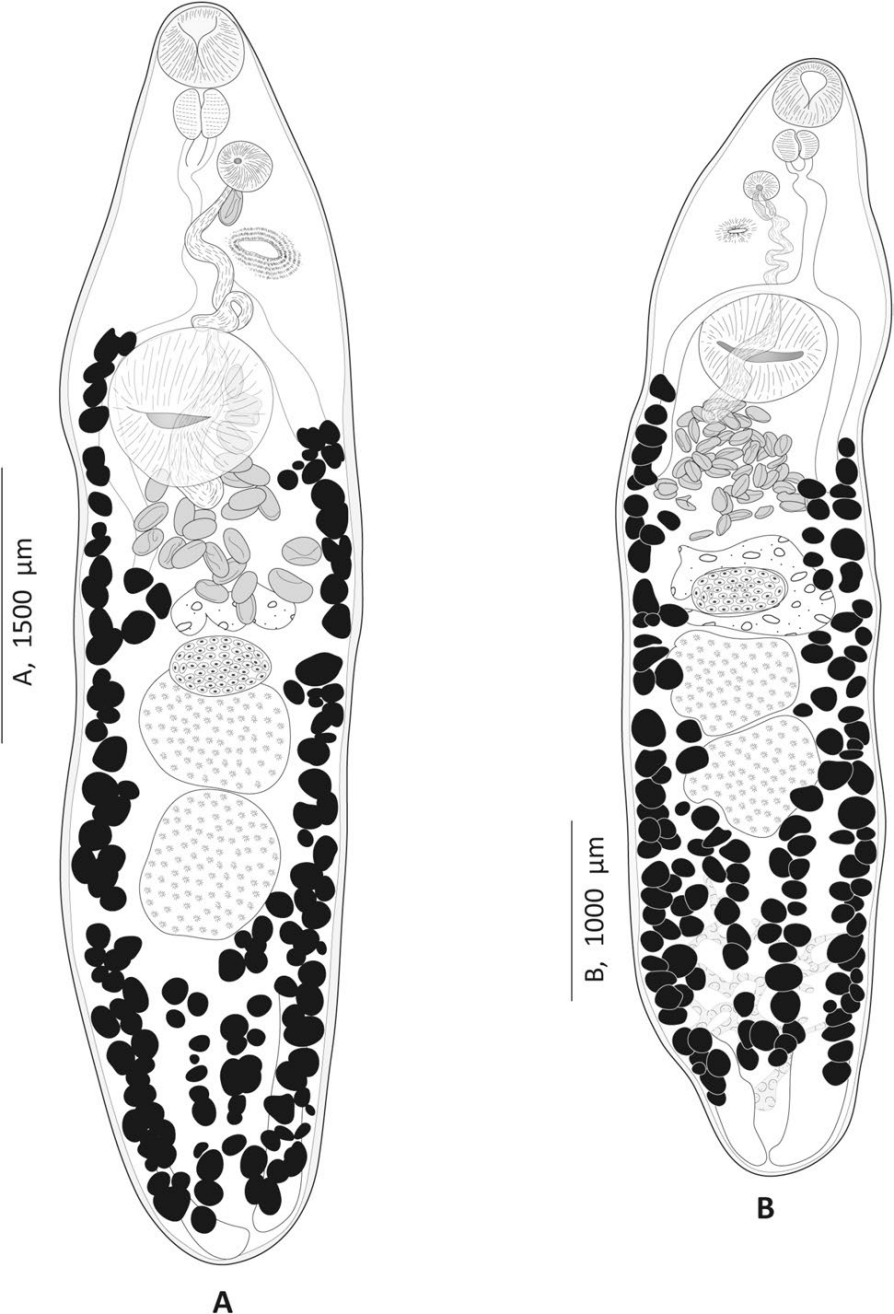
*Genitocotyle necromnemos* n. sp. from the intestine of *Apogon imberbis*, off Algeria. **A** Body, ventral view. **B** Body, dorsal view

**Fig. 2:**
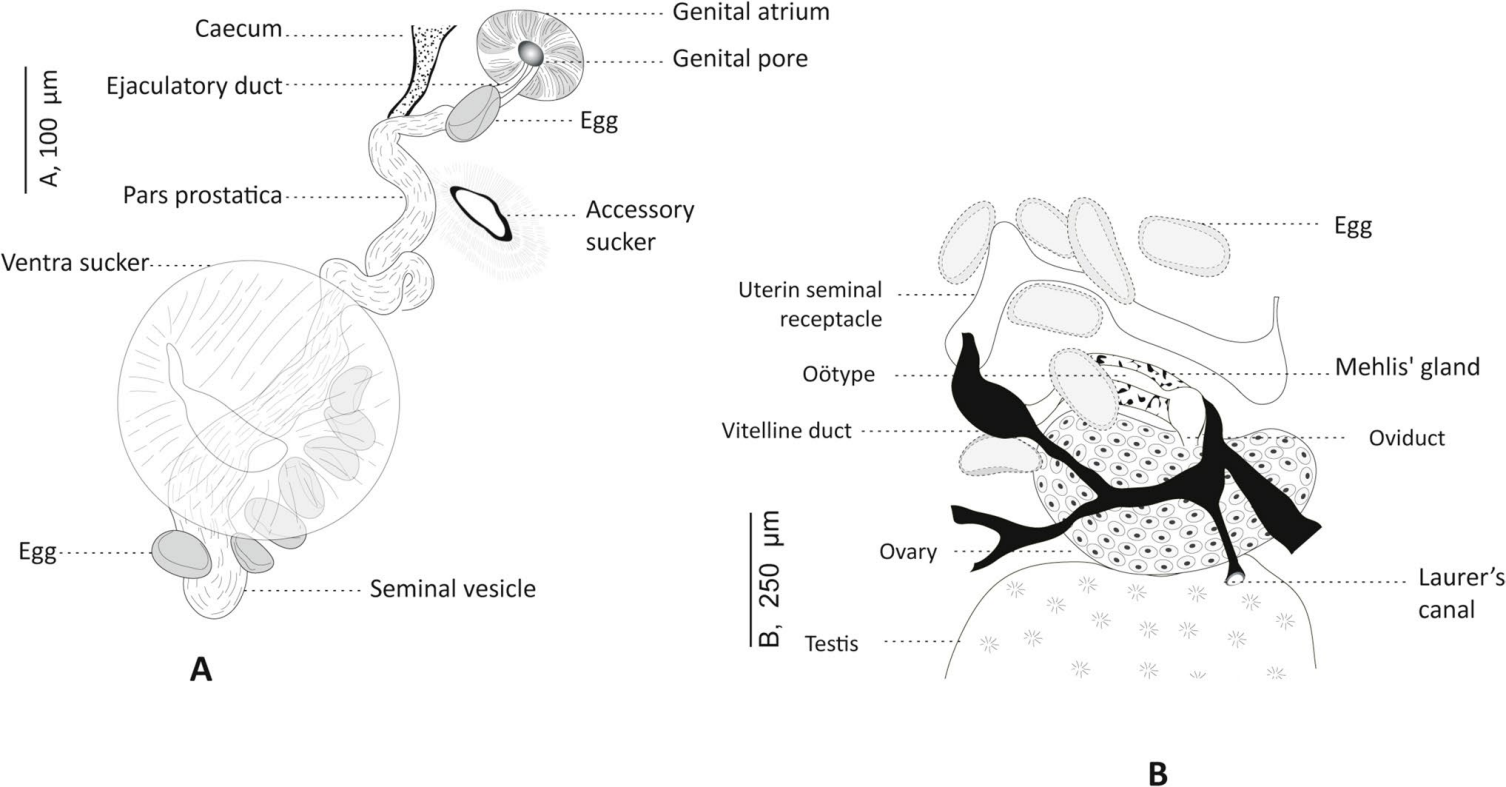
*Genitocotyle necromnemos* n. sp. from the intestine of *Apogon imberbis*, off Algeria. **A** Ventral view of terminal genitalia. **B** Dorsal view of the female reproductive system

**Fig. 3:**
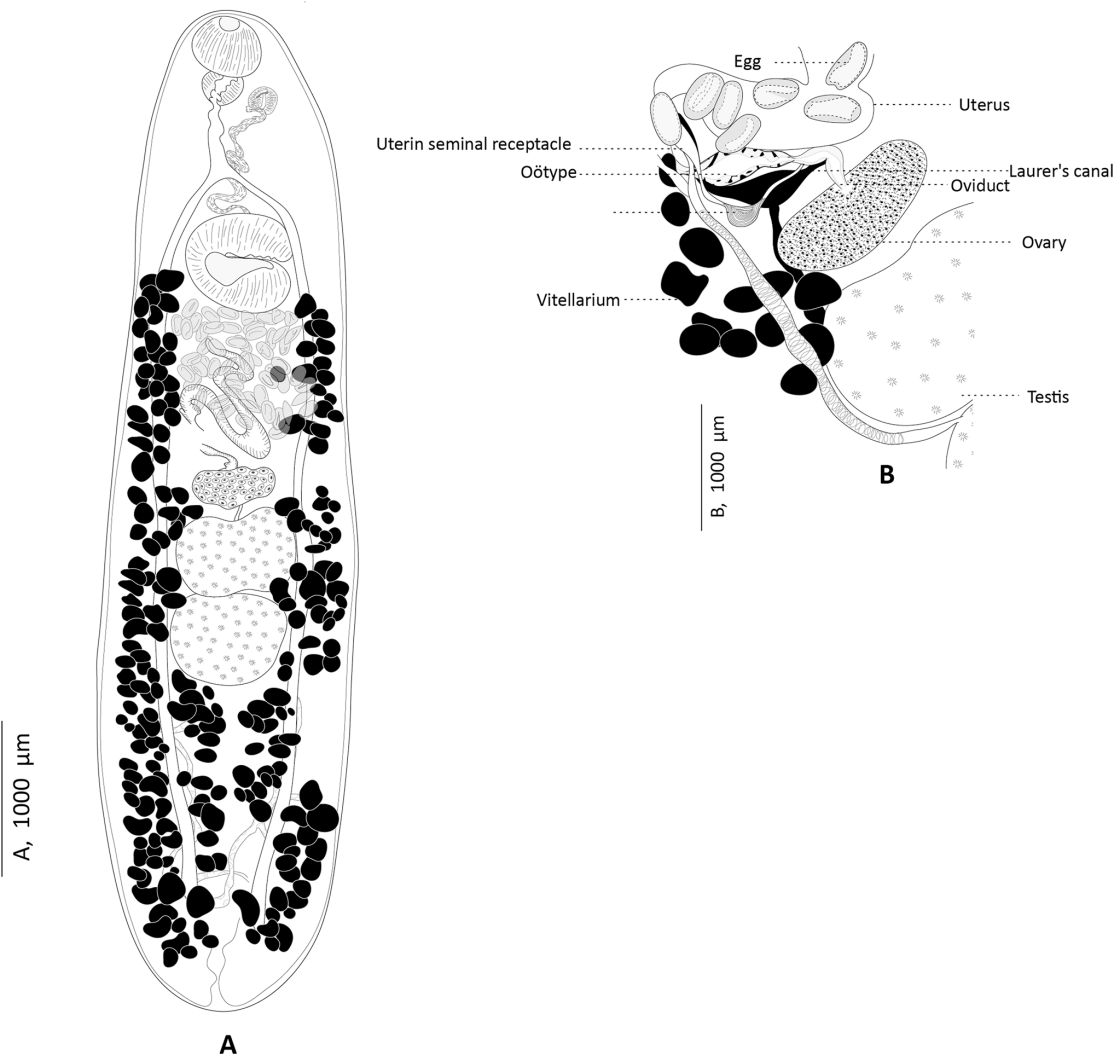
*Genitocotyle necromnemos* n. sp. from the intestine of *Apogon imberbis*, off Madeira, historical specimens from the Natural History Museum (NHM 1980.7.17. 228). **A** Body, dorsal view. **B** Ventral view of the female reproductive system

*Genitocotyle necromnemos* n. sp. (Figs. 1, 2, 3)

*Synonyms*: *Genitocotyle* sp. *sensu* Bartoli, Gibson [10].

*Type-host*: *Apogon imberbis* (Kurtiformes: Apogonidae), the cardinal fish.

*Type-locality*: Dellys, 36.912984° N, 3.928383° E, Algeria, Western Mediterranean.

*Other localities*: Tamentfoust, 36.803675° N, 3.231279° E, Algeria, Western Mediterranean and Madeira, Eastern Central Atlantic [10].

*Site in host*: Intestine.

*Infection details*: Prevalence: 21.6 %; mean abundance: 0.22.; mean intensity: 3.04.

*Type specimens*: Holotype (SMNH Type-9988) and five paratypes (SMNH Type-9989–9993) deposited in the Type collections of the Swedish Museum of Natural History (SMNH), Stockholm, Sweden. 10 paratypes (LBEIG-ApGn-L7-ApGn-L16) deposited in the collections of Laboratoire de Biodiversité et Environnement, Interactions—Génomes, Université des Sciences et de la Technologie Houari Boumediene (USTHB), Algiers, Algeria.

*Vouchers*: Two specimens of *Genitocotyle* sp. ex *Apogon imberbis* off Madeira [NHMUK Coll. No. 1980.7.17.228].

*ZooBank registration*: urn:lsid:zoobank.org:pub:B2E79107-1C21-4A27-9EC1-2453C9DA42A2.

*Etymology*: The species name “*necromnemos*” is derived from two Greek words: *necro*-, meaning “dead” or “from death,” referencing the long-standing mystery surrounding these specimens that originated off Madeira and died in 1951 in the aquarium of London Zoo) and preserved the Natural History Museum (London); and -*mnemos*, meaning"memory"alluding to the historical nature of these specimens, which had remained undescribed and largely forgotten until their rediscovery and formal description in this study.

### Description

[Based on 20 whole- flattened gravid specimens; measurements in Table 1].

**Table 1:** Morphomotry of *Genitocotyle* species. ^1^ Two specimens, previously annotated as *Genitocotyle* sp. from *Apogon imberbis* off Madeira, from the Natural History Museum. London [NHM 1980. 7. 17. 228]. ^2^ Other hosts are *Carapus bermudensis* (Carapidae), *Malacoctenus macropus*, (Labrisomidae), *Opistognathus* sp. (Opistognathidae), *Syngnathus robertsi* (Syngnathidae) (Manter, 1947)

	*G. necromnemos* n. sp.		*G. mediterranea*	*G. cablei*	*G. heterostichi*	*G. acirrus*	*G. atlantica*
Host	*Apogon imberbis* (Apogonidae)		*Symphodus ocellatus* (Labridae)	*Ancylopsetta quadrocellata* (Paralichthyidae)	*Heterostichus rostratus* (Clinidae)	*Amphistichus rhodoterus* (Embiotocidae)	*Haemulon* *flavolineaatum*^2^ (Haemulidae)
Locality	Dellys, Tamentfoust Algeria, WM	Maderia, Portugal, NEA	Corsica, France, WM	Dog Island Reef, WCA	La Jolla, California, ECP	Dillon’s Beach, California, ECP	Tortugas, Florida, WCA
No. of specimens	20	2	10	2	10	30	
Reference.	Present study	Present study^1^	Bartoli *et al.* [10]	Nahhas and Short [14]	Montgomery [15]	Park [12]	Manter [13]
Body L.	1121–3408 (1994, n = 20)	1939, 2425	746–2201 (1307)	2700–2930	1190–1730	2580 (2030–3480)	892–1530
Body W.	222–723 (407, n = 20)	457, 500	213–421 (284)	567–600	170–280	8700 (7500–1130)	280–345
Forebody	280–789 (450, n = 20)	400, 410	266–576 (364)	–	310–530	–	
Hindbody	658–2378 (1337, n = 20)	1310, 1790	522–1396 (816)	–	–	–	–
Oral sucker L.	81–152 (117, n = 20)	130, 133	74–108 (89)	165–185*	80–90	2000 (1700–2400)	0.064–0.1 64–120
Oral sucker W.	75–174 (119, n = 20)	140, 173	80–128 (101)		70–90		
Ventral sucker L.	149–353 (221, n = 20)	210, 254	130–217 (170)	268–294*	140–170	3700 (3400–4200)	96–200
Ventral sucker W.	141–334 (219, n = 20)	230, 269	130–217 (169)		140–170		
Prepharynx L.	9–27 (16, n = 15)		11–39 (17)				
Accessory sucker L.	27–79 (37, n = 20)	90, 83	43–98 (64)		70–79*	1800 (1600–1900)	
Accessory sucker W.	7–60 (42, n =20)	100, 95					
Pharynx L.	51–147 (74, n = 20)	80, 80	46–72 (55)	155*	30–60	1400 (110–180)	48–54
Pharynx W.	49–128 (82, n = 20)	78, 80	56–93 (71)		50		43–48
Oesophagus L.	85–276 (186, n = 15)	–	91–228 (129)	294–360	4–5 times pharynx L.	260 (150–380)	
Genital atrium		–	30–80 (51)				
Anterior testis L.	85–374 (192, n = 20)	129, 145	98–228 (144)	232–309*		210 (140–300)	
Anterior testis W.	118–470 (246, n = 20)	222, 240	130–304 (203)			360 (290–530)	
Posterior tests L.	79–401 (206, n = 20)	147, 198	98–250 (160)			290 (170–410)	
Posterior tests W.	126–497 (244, n = 20)	231, 218	130–271 (193)			360 (230–470)	
Ovary L.	48–213 (93, n = 20)	75, 79	50–108 (74)	155–180*		140 (60–200)	
Ovary W.	61–250 (147, n = 20)	162, 197	102–228 (152)			260 (200–380)	
Number of egg			7–39 (18)		6–12		
Egg L.	41–61 (52, n = 18)	46, 50	49–59 (53)	56–64	66–70	70–80	52–58
Egg W.	20–45 (30, n = 18)	28, 29	24–32 (28)	31–36	45–55	30–40	26–30
Ventral sucker to ovary	142–644 (311, n = 20)	310, 450	109–325 (169)				
Ventral sucker to anterior testis	190–863 (414, n = 20)	400, 550	130–434 (239)				
Post–testicular L.	262–856 (511, n = 20)	620, 840	174–477 (279)		510–1020		
Sucker-ratio	1:0.53–0.52 (0.51)		1:1.67–2.12 (1.91)	1:1.54–1.62	1:1.8–1.91		1:1.45–1.8 (1.5)
Oral sucker/pharynx ratio	1:1.53–1.35 (0.34)		1:0.53–0.69 (0.62)				
Forebody/hindbody ratio	1:0.42–0.26 (0.34)		1:1.71–2.54 (2.20)				
Forebody, L. of body ratio	1:0.25–0.23 (0.24)		1:3.2–4 (3.7)				
Forebody/Body L. (%)	25–24% (23; n = 20)		36–26 (28%)		26–31%		1/4 body L. (25%)
Hindbody/Body L. (%)	59–70% (67; n = 20)		70–63 (62%)		–		–
Ventral sucker to ovary/Body L.	13–19% (16; n = 20)		14–15 (13%)				
Ventral sucker to anterior testis/Body L. (%)	17–25% (21; n = 20)		17–20 (18%)				
Post–testicular L./Body L. (%)	23–25% (26; n = 20)		23–22 (21%)		43–59 (51%)		

All Measurements are in micrometres and indicated as the range followed by mean values in parentheses for some species

*WM* Western Mediterranean, *NEA* Northeast Atlantic, *WCA* Western-Central Atlantic, *ECP* Eastern Central Pacific, *L* Length, *W* Width

*Diameter

Body elongate, dorsoventrally flattened, widest at level of ventral sucker, rounded posteriorly. Forebody and hindbody occupying 23–25 (24; n = 20) and 59–70 (64; n = 20) % of body length, respectively. Post-testicular region shorter than forebody. Tegument thick, unarmed. Oral sucker ventro-subterminal. Ventral sucker located at limit of anterior and middle thirds of body, rounded, larger than oral sucker; ventral sucker to oral sucker length ratio 2:1.8–2.3 (2; n = 20), width ratio 1:1.87–1.91 (1.89; n = 20).

Accessory sucker present, without a limiting membrane, approximately midway between genital atrium and ventral sucker; aperture transverse, elliptical with visible radiating muscles. Pre-pharynx short. Pharynx large, ovoid. Esophagus long, thin-walled, bifurcating at level of anterior margin of ventral sucker. Caeca thick-walled, terminating blindly near posterior end of body.

Testes 2, tandem, entire, post-ovarian and located in mid-hindbody. Cirrus-sac absent. *Vasa efferentia* uniting to form *vas deferens* at base of seminal vesicle. Seminal vesicle long, tubular, oval or contoured, extending posteriorly to ventral sucker. Pars prostatica cylindrical. Genital atrium spherical, thick-walled, halfway between pre-pharynx and accessory sucker. Ejaculatory duct long, opening into genital atrium.

Ovary median, unlobed, pre-testicular, transversely elongated. Oviduct short. Laurer's canal connected to oviduct, opening dorsally to ovary. Oötype voluminous and surrounded by Mehlis’ gland, pre-ovarian. Canalicular seminal receptacle absent. Seminal receptacle uterine present. Uterus pre-ovarian, sinuous, extending to anterior edge of ventral sucker, ending with metraterm; metraterm thick-walled, opening into genital atrium. Eggs ovoid, few in numbers in uterus (around 30). Vitellarium follicular, vitelline follicles numerous and voluminous; ventrally, vitelline fields extending from anterior edge of acetabulum to posterior end of body, merging in post-testicular area; dorsally, vitelline fields extending from posterior edge of ventral sucker to two thirds of post-testicular area; transverse vitelline ducts situated in post-testicular region.

### Remark

Two specimens of *Genitocotyle* sp. collected from *A. imberbis* off Madeira (see above) show similarities with *G. mediterranea* as noted by Bartoli and Gibson [10], but show notable differences, such as larger size, more numerous eggs in the uterus, a longer post-testicular zone and a seminal vesicle apparently restricted to the forebody. In this study, we re-examined these two specimens, and our analysis presented through illustrations and measurements confirms their conspecificity (Fig. 3; Table 1). Notably, both populations of *Genitocotyle* ex *A. imberbis* (museum specimens off Madeira and newly collected specimens off Algeria) share the extent vitellarium, supporting their conspecificity and classification as the same species, described here as *G. necromnemos* n. sp.

## Discussion

To date, there are four nominal species of *Genitocotyle* : *G. acirrus* and *G. heterostichi*, occur in the Eastern Central Pacific [12, 18, 25, 26]; *G. cablei* and *G. atlantica* occurring in the Western-Central Atlantic [13, 17, 19, 27]; and *G. mediterranea* known only from Mediterranean waters [10]. Morphologically, *G. necromnemos* n. sp. ex *A. imberbis* off Algeria can be distinguished from *G. acirrus* in that the vitelline follicles extend into the forebody (vs. not surpassing the level of ventral sucker) and by the possession of an unlobed ovary (vs. lobed, with three lobes). *Genitocotyle necromnemos* n. sp. is readily distinguished from *G. atlantica* in having an unlobed ovary (vs. distinctly bilobed ovary in *G. atlantica*). Additionally, *G. atlantica* is readily differentiated from *G. necromnemos* n. sp. by the testes being wider than they are long and their outline is irregular or lobed, the post-testicular distance being approximately equal to or slightly greater than forebody length, and by having an ovoid ventral sucker. *Genitocotyle cablei* is similar to *G. necromnemos* n. sp. in that the ovary is unlobed, but differs in the extension of vitelline follicles (from the posterior end of the body to the posterior margin of the ventral sucker in *G. cablei *vs. extending to the anterior margin of the ventral sucker in *G. necromnemos* n. sp.), and by the post-testicular distance being longer than the length of the forebody in *G. cablei*. *Genitocotyle heterostichi* is easily distinguished from *Genitocotyle necromnemos* n. sp. in that the vitelline follicles reach the anterior margin of the posterior testis, an ovary with three lobes of ovoid shape, in having a greater post-testicular length, about 1/2 of the body, greater than forebody length, and by the seminal vesicle that does not extend posteriorly to the acetabulum.

*Genitocotyle mediterranea* resembles *G. necromnemos* n. sp. in that the vitelline follicles extend from the posterior end of the body to the anterior margin of the ventral sucker. *Genitocotyle mediterranea* differs from *G. necromnemos* n. sp. in having an ovary distinctly or indistinctly three-lobed (vs. unlobed in *G. necromnemos* n. sp.), having a transversely elongate ovary; median lobe posteriorly oriented and post-testicular length, about 1/4 of the body, less than the length of the forebody.

**Fig. 4:**
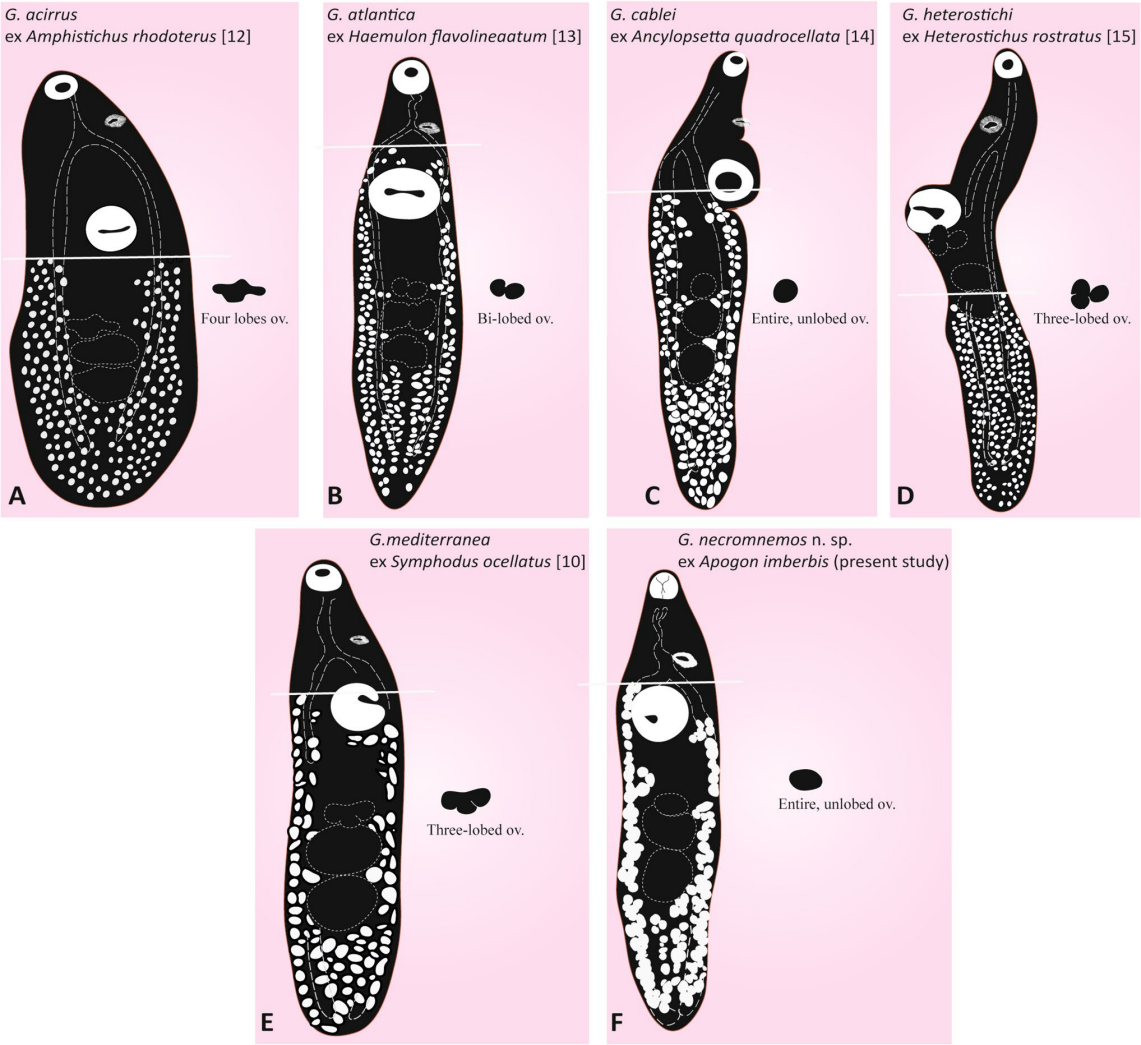
Diagram showing the differences among *Genitocotyle* species in distribution of the vitelline follicles and shape of the ovary. A, *Genitocotyle acirrus* Park, 1937 ex *Amphistichus rhodoterus* [12]. B, *Genitocotyle atlantica* Manter, 1947 ex *Haemulon flavolineaatum* [13]. C, *Genitocotyle cablei* Nahhas and Short, 1965 ex *Ancylopsetta quadrocellata *[14]. D, *Genitocotyle heterostichi *ex *Heterostichus rostratu*s Montgomery, 1957 [15]. E, *Genitocotyle mediterranea* Bartoli, Gibson and Riutort, 1994 ex *Symphodus ocellatus* [10]. F, *Genitocotyle necromnemos* n. sp. ex *Apogon imberbis* (present study)

A comparative diagram of six *Genitocotyle* species (Fig. 4) suggests that the distribution of the vitelline follicles is a useful character to distinguish *Genitocotyle* species.

In *G. acirrus*, the vitelline follicles reach as far as the posterior edge of the ventral sucker (Fig. 4A). In *G. atlantica*, the vitelline follicles extend to the intestinal bifurcation (Fig. 4B). In *G. cablei*, the vitelline follicles reach the posterior edge of the ventral sucker (Fig. 4C). In *G. heterostichi*, the vitelline follicles extend to the posterior testis (Fig. 4D). In *G. mediterranea*, the vitelline follicles extend to the anterior edge of the acetabulum (Fig. 4E). In *G. necromnemos* n. sp., the vitelline follicles extend to the intestinal bifurcation (Fig. 4F).

We also tried to test the usefulness of the organization and shape of the ovary, and in *G. acirrus*, the ovary is clearly or indistinctly four-lobed (Fig. 4A). In *G. atlantica*, the ovary is bi-lobed (Fig. 4B). In *G. cablei*, the ovary is rounded and not lobed (Fig. 4C). In *G. heterostichi*, the ovary is ovoid with three lobes (Fig. 4D). In *G. mediterranea*, the ovary is distinctly or indistinctly three-lobed (Fig. 4E). In G. *necromnemos* n. sp., the ovary is ovoid and unlobed (Fig. 4F). These differences in the extent of the vitelline follicles and the morphology of the ovary highlight the usefulness of these features in distinguishing among *Genitocotyle* spp.

## Conclusion

**Fig. 5:**
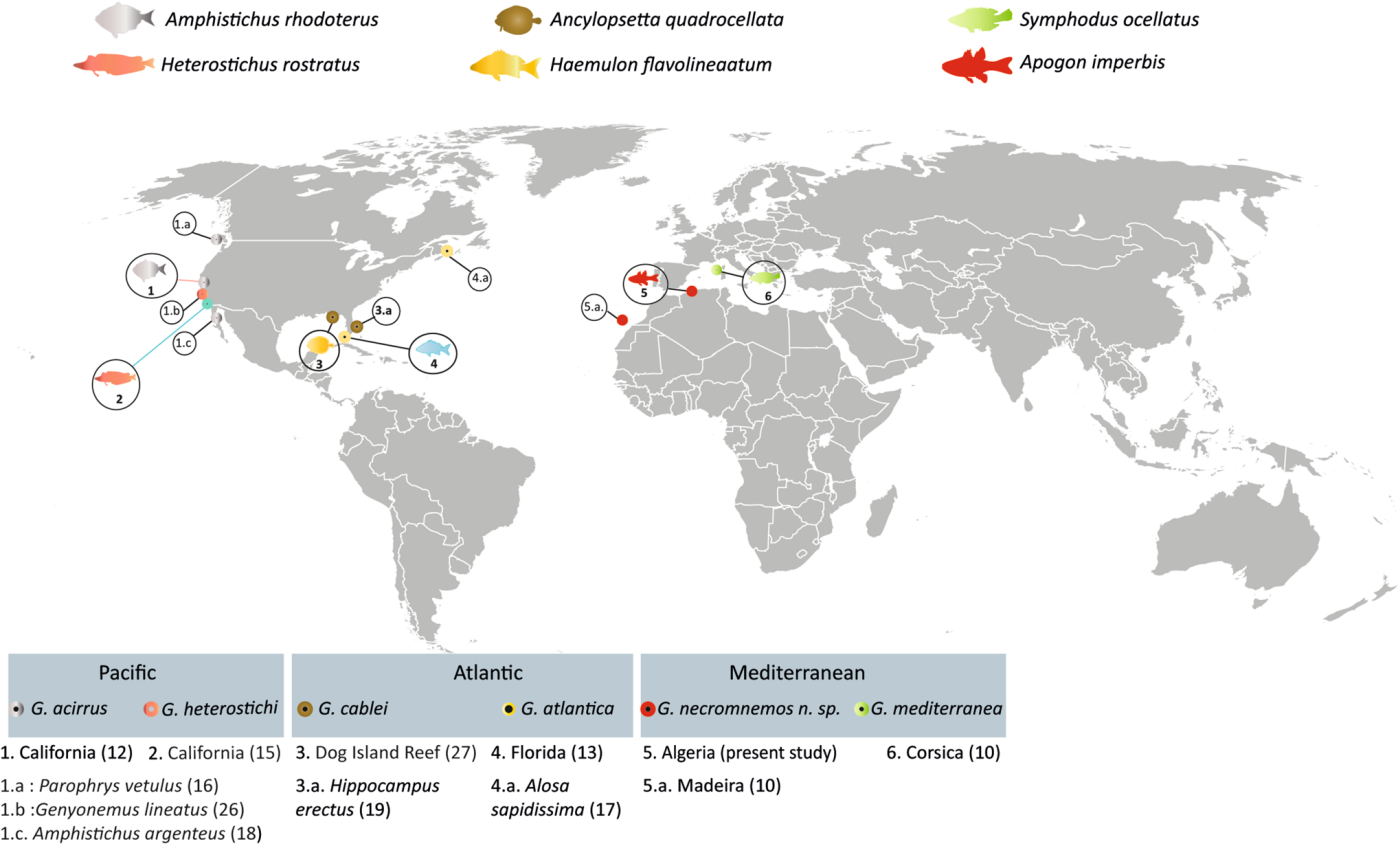
Geographic distribution of *Genitocotyle* species including the type hosts and type localities. Numbers in bold indicate type hosts mentioned in the original descriptions; numbers followed by letters represent secondary records. All references for the records are given in parentheses

In this study, we described *G. necromnemos* n. sp., a new species of digenean based on the re-examination of museum specimens and freshly collected material from *Apogon imberbis* off Algeria. Our findings confirm that this species is distinct from other members of the genus *Genitocotyle* based on key morphological features, including the unlobed ovary and the extension of the vitelline follicles. The comparative analysis of these specimens, alongside other described species, highlights the importance of the distribution of the vitelline follicles and the organization of the ovary as distinguishing characters within the genus. This study also confirms *G. necromnemos* n. sp. as the first *Genitocotyle* species described from an apogonid host and provides a clearer understanding of the diversity within this genus. Further research on the ecological and zoogeographical aspects of this species will contribute to a more comprehensive understanding of *Genitocotyle* species’ host specificity and distribution (Fig. 5).

## Data Availability

No datasets were generated or analysed during the current study.
